# The nuclear factor-κB correlates with increased expression of interleukin-6 and promotes progression of gastric carcinoma

**DOI:** 10.3892/or.2012.2089

**Published:** 2012-10-19

**Authors:** YEFENG YIN, XIULIAN SI, YAN GAO, LEI GAO, JIANGNING WANG

**Affiliations:** Department of Plastic and Reconstruction Surgery, Beijing Luhe Hospital, Capital Medical University, Beijing 101199, P.R. China

**Keywords:** gastric cancer, interleukin-6, nuclear factor-κB, vascular endothelial growth factor

## Abstract

The interleukin-6 (IL-6) pathway is one of the mechanisms that link inflammation and angiogenesis with malignancy. Since nuclear factor-κB (NF-κB) is a potential sign for inflammation, NF-κB has been associated with the progression of disease in various types of cancer. In the present study, we investigated the effect of NF-κB on the IL-6 pathway in gastric carcinoma and their correlation with disease status and prognosis. The mRNA and protein levels of NF-κB, IL-6 and vascular endothelial growth factor (VEGF) were detected by western blotting and reverse transcription (RT) quantitative PCR (RT-qPCR). Using immunohistochemistry, we examined the expression of these proteins in normal and human gastric cancer tissue samples. The concentrations of IL-6 and TNF-α in collected blood samples were measured according to the enzyme-linked immunosorbent assay (ELISA). IL-6 and TNF-α were found to be expressed at high levels in human gastric cancer samples. A positive correlation was found between the expression of IL-6 and NF-κB by immunohistochemical and further correlation analysis. IL-6, NF-κB and VEGF protein and mRNA levels increased significantly in gastric cancer tissue compared with those in adjacent normal mucosa tissue samples. In conclusion, our findings demonstrate that NF-κB, IL-6 and VEGF mRNA and protein levels increase significantly in gastric cancer tissues. In addition, the expression of NF-κB was positively correlated with the expression of IL-6 according to immunohistochemical and further correlation analysis, which suggests that the suppression of NF-κB or IL-6 may be a potential target for clinical therapy of gastric cancer in the future.

## Introduction

Interleukin-6 (IL-6) is a pleiotropic inflammatory cytokine that induces the growth and differentiation of immune cells as well as the expression of many cytokines. IL-6 is also a representive marker of clinical correlation and prognostic factor in patients with cancer (1,2). Angiogenesis is an essential process in the progression and development of cancer. The association of IL-6 with angiogenesis depends on its ability to induce the production of vascular endothelial growth factor (VEGF), which is a very potent angiogenic agent (1). Additionally, IL-6 activates the RhoA and phosphorylated-Src protein, which is associated with aggressive lymph node metastasis and poor survival in malignancy (3).

Nuclear factor-κB (NF-κB), a nuclear protein, was first identified as a transcription factor in the nuclei of mature B lymphocytes (4). It regulates the expression of various genes, particularly those involved in the inflammatory and immune responses (5). Recent evidence has revealed that the activity of the NF-κB pathway is significantly involved in the process leading from inflammation to carcinogenesis and tumor development (6). NF-κB promotes the overexpression of inflammatory cytokines that act as tumor growth factors for colitis-associated cancer (7). IL-6, which is encoded by an NF-κB target gene, is proposed to be one of these tumor growth factors. Specific inactivation of IL-6 signaling by antagonistic anti-IL-6 antibodies inhibited tumor growth, similar to the inhibition of TGF-β signaling in colorectal cancer (8). Furthermore, progression and chemoresistance also appear to involve IL-6, NF-κB induced expression of IL-6 by its regulation of the growth and survival of tumor cells (9,10).

Gastric carcinoma is the fourth most frequent malignancy worldwide and the second most common cause of mortality; it is the result of accumulated genomic damage which is crucial for cancer development (11,12). The high rates of gastric cancer mortality may be related to direct invasion into the adjacent organs, lymph node metastasis, and distant metastasis of gastric cancer. IL-6 plays a positive role as a prognostic factor in lymph node metastasis and advanced gastric cancer (13). However, whether the expression of IL-6 correlates with the expression of NF-κB in patients suffering from gastric cancer remains unclear. The aim of our study was to investigate the protein and mRNA levels of IL-6 and NF-κB and to analyze the correlation of these two proteins in gastric cancer patients.

## Materials and methods

### Patients

Eligible patients were adults (18–75 years old), who had been diagnosed with biopsy-confirmed gastric cancer. Fresh cancer tissue samples and corresponding normal tissue samples from areas adjacent to the tumor specimens (≥5 cm) were obtained from the patients. All patients were screened and treated at Beijing Luhe Hospital and samples for the current study were obtained with the informed consent of the patients. Each tissue fragment was divided into three parts; one portion was processed for immunohistochemistry, the second portion for western blot analysis freezing them in liquid nitrogen, and the third portion was for reverse transcription (RT) quantitative PCR (RT-qPCR), freezing them in liquid nitrogen.

### Determination of serum cytokines

All blood samples without EDTA were centrifuged at 100,000 rpm for 15 min at 4˚C immediately, and the supernatant was all stored at −80˚C until analysis. Enzyme-linked immunosorbent assay (ELISA) (R&D Systems, USA) was used to detected the serum level of human TNF-α, IL-6, according to the manufacturer’s instructions.

### Immunohistochemistry

Sections (5 μm) of formalin-fixed, paraffin-embedded primary gastric specimens were prepared for immunohistochemical analysis. The sections were stained with antibody (Santa Cruz Biotechnology, USA). The expression levels of VEGF, NF-κB, and IL-6 in the experimental gastric samples were determined by an anti-VEGF antibody (1:100 dilution), an anti-NF-κB antibody (1:100 dilution) and an anti-IL-6 antibody (1:50 dilution). Non-specific IgG antibody was used for negative control of tissue sections. Specific antibody staining was visualized using a diaminobenzidine substrate kit. All slides were observed under a bright-field microscope.

### Reverse transcription quantitative PCR

Samples (including gastric cancer tissue and the tissue of corresponding normal areas) were treated with the TRIzol reagent (Invitrogen, USA) for total-RNA extraction. The potentially contaminated genomic DNA was removed by treating 10 mg of the RNA sample at 37˚C for 30 min with 1 ml of TURBO DNase (Ambion, USA) followed by extraction with phenol:chloroform:isoamyl alcohol (25:24:1). Real-time PCR analysis was carried out on the ABI PrismH 7300 Sequence Detection System (Applied Biosystems, USA). Expression of IL-6, NF-κB and VEGF were analyzed using the TaqMan PCR Master Mix Reagents kit (Applied Biosystems). The TaqMan probe and primers for human IL-6, NF-κB and VEGF designed using the Primer Express 2.0 version were: NF-κB forward 5′-gaaccacacccctgcatatag-3′, reverse 5′-gcattttcccaagagtcatcc-3′ and probe 5′-agaggcta aagttctccaccagg-3′; IL-6 forward 5′-ccactcacctcttcagaacg-3′, reverse 5′-catctttggaaggttcaggttg-3′ and probe 5′-aaattcggta catcctcgacggcatc-3′; VEGF forward 5′-agtccaacatcaccatgcag-3′, reverse 5′-ttccctttcctcgaactgattt-3′ and probe 5′-tcaccaaggccag cacataggag-3′. The cDNA was synthesized from 500 ng of RNA using the TaqMan RT Reagents kit (Applied Biosystems). The optimized concentrations for real-time PCR were 0.4 μM for both primers, 0.2 μM for the probe and 5 ng cDNA in a 20 μl reaction volume. Human actin primers (forward 5′-tgcagaaag agatcaccgc-3′, reverse 5′-ccgatccacaccgagtatttg-3′) were used as an internal control. Each sample was tested in triplicate. Cycle threshold (Ct) values were obtained graphically for IL-6, NF-κB, VEGF and actin. The difference in Ct values between actin and IL-6, NF-κB, VEGF are presented as ΔCt values. The ΔΔCt values were obtained by subtracting the ΔCt values of the control samples from those of the treated samples. Relative fold change in gene expression was calculated as 2^−ΔΔCt^.

### Western blot analysis

Whole tissue lysates were prepared from human gastric tissue specimens. Standard western blotting was performed using anti-IL-6 and anti-NF-κB, anti-VEGF antibodies (Santa Cruz Biotechnology). Simultaneous determination of the expression level of β-actin was carried out as an internal control. Proteins were detected using the enhanced chemiluminescence system in accordance with the manufacturer’s instructions (Tanon 4500, Shandong Aibo Technology Co., China). Separate analyses were performed for each sample and the experiment was repeated three times.

### Statistical analysis

Data were expressed as the mean ± SD. Values were performed with a one-way analysis using SPSS15.0 software, followed by a student’s two-tailed test, and comparison between groups was performed using an analysis of variance (ANOVA) or through a non-parametric test. P<0.05 was considered to indicate statistically significant differences.

## Results

### Determination of serum samples quantifying IL-6, TNF-α

Ninety-eight patients were enrolled in this study. Patient plasma samples were collected to determine cytokine levels prior to and following surgery. IL-6 and TNF-α were examined to conform any differences in plasma pre-and post-operatively. Cytokine concentration (P<0.005) of IL-6, TNF-α decreased in post-operative plasma samples (Table I). Two-paired t-test was used by observing a significant difference in pre-operative, post-operative and normal serum samples.

### Immunohistochemical expression and cellular distribution of IL-6, NF-κB, and VEGF

**Figure 1:**
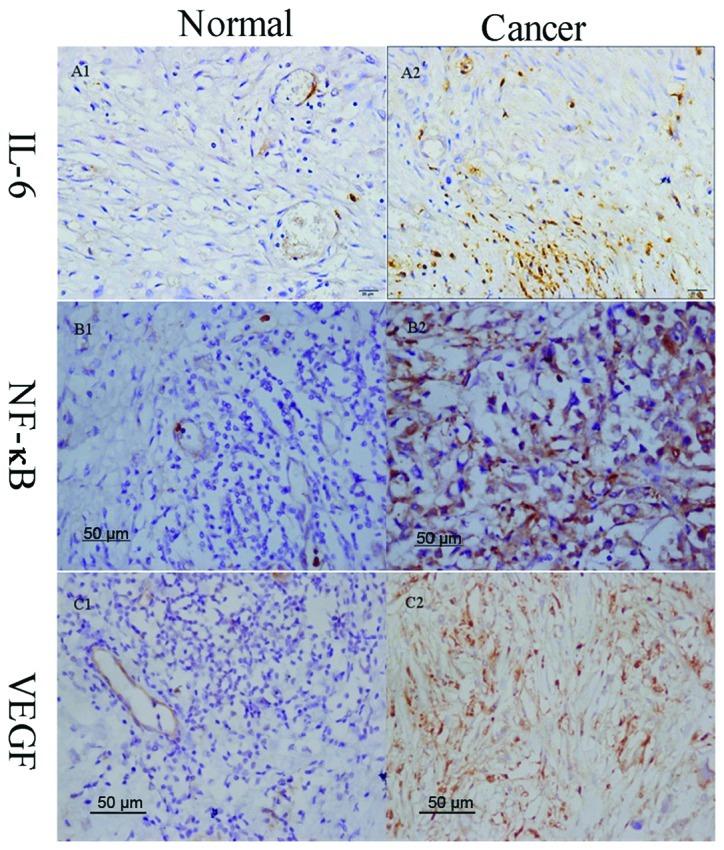
Immunostaining showed that NF-κB activity is directly associated with protein overexpression in human gastric cancer tissue. There was a weak expression of NF-κB (B1) in adjacent normal mucosa but a strong expression of NF-κB in gastric cancer tissue. All NF-κB was localized in the nuclei of the tumor cell and was performed by numerous yellowish granules (B2). IL-6 (A1 and A2) and VEGF (C1 and C2) positive cells increased significantly in gastric cancer tissue.

**Figure 2:**
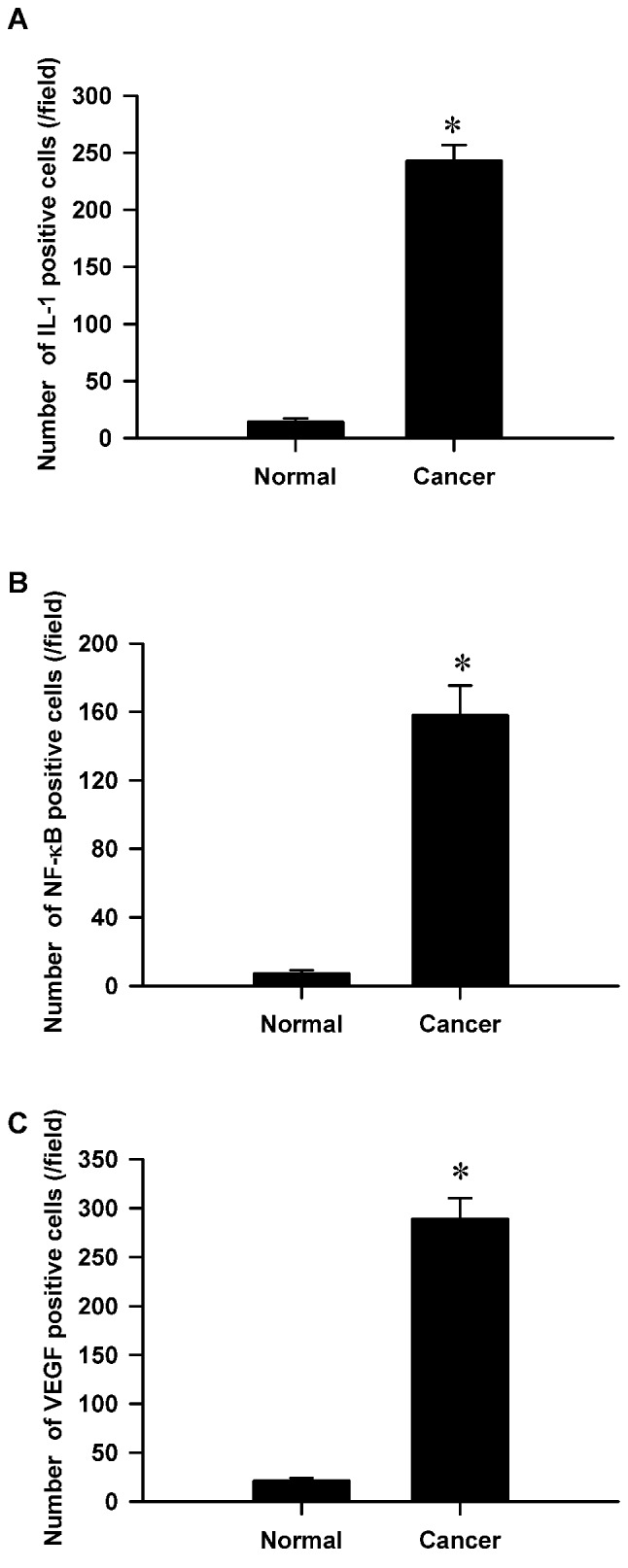
IL-6 (A), NF-κB (B) and VEGF (C) positive cells were counted by the Olympus Denmark A/S (Denmark) CAST-Grid system in adjacent normal mucosa and gastric cancer tissue samples. ^*^P<0.05 compared with normal tissue, n=20.

**Figure 3:**
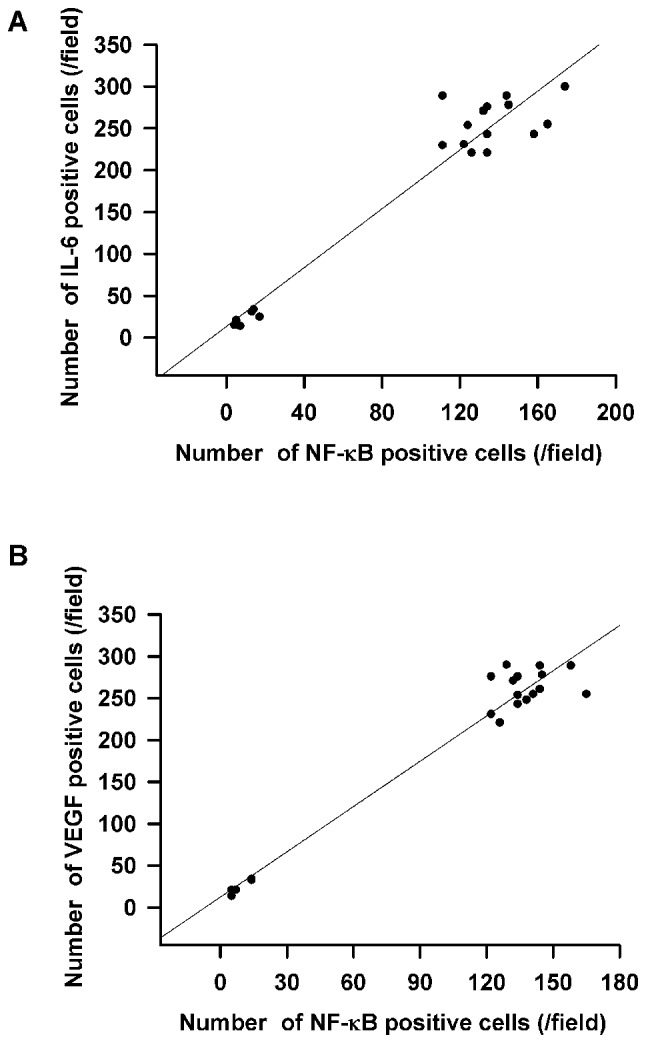
Further correlation analysis was conducted according to the number of IL-6, NF-κB and VEGF positive cells (per field) in normal mucosa and gastric cancer tissue samples of 27 different patients (A and B).

The production of IL-6, NF-κB and VEGF in human gastric tissue and adjacent normal mucosa were all examined using immunohistochemical staining. The findings of the immunohistochemical staining confirmed a weak expression of NF-κB, IL-6 and VEGF in adjacent normal mucosa but a strong expression in gastric cancer tissue (Figs. 1 and 2A–C). The overexpression of IL-6 was directly associated with NF-κB activation (Fig. 3A). Overexpression of VEGF was also directly associated with NF-κB activation according to further correlation analysis (Fig. 3B). Moreover, we found an association of increased IL-6, VEGF and NF-κB expression in the clinicopathological characteristics of gastric cancer (Fig. 3A and B).

### mRNA levels are significantly increased in gastric cancer tissue

**Figure 4:**
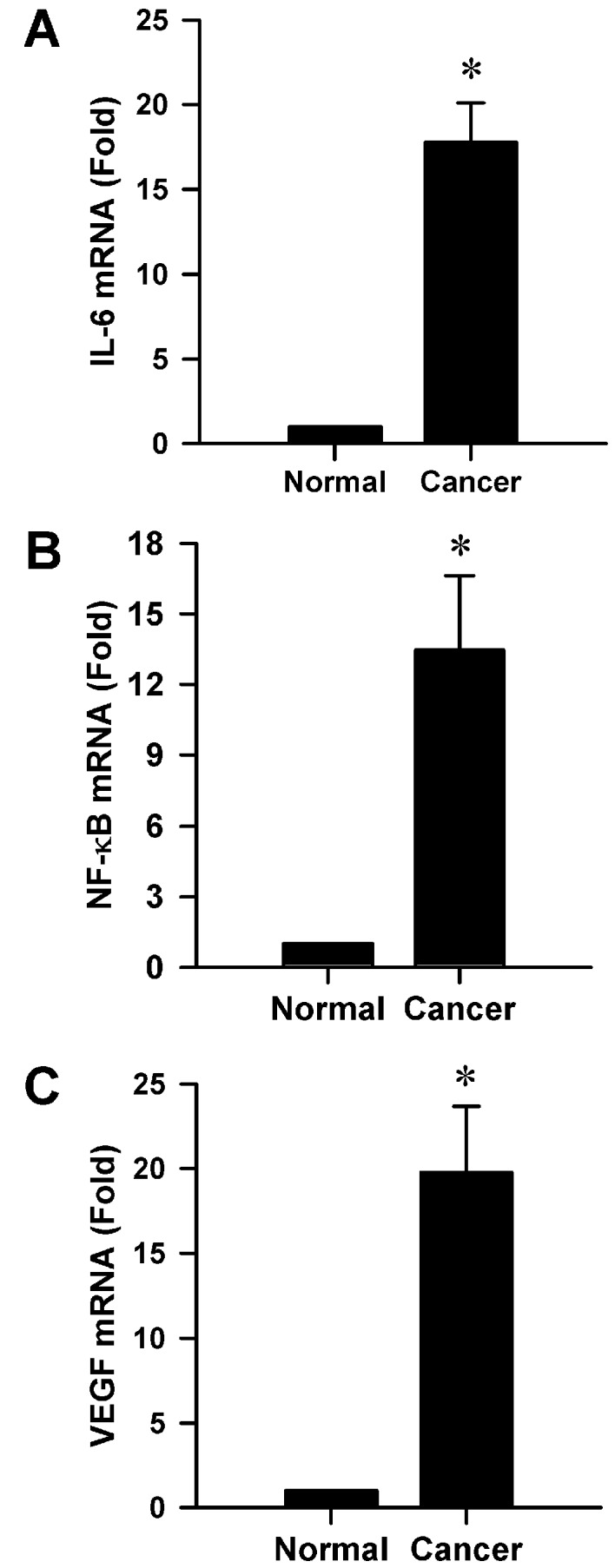
mRNA expression of IL-6 (A), NF-κB (B), and VEGF (C) was determined by RT-qPCR in adjacent normal mucosa and gastric cancer tissue samples. ^*^P<0.05, compared with normal tissue, n=6.

We investigated the mRNA levels of IL-6, NF-κB and VEGF in gastric cancer tissue according to RT-qPCR. As we expected, mRNA levels of IL-6, NF-κB and VEGF in human gastric cancer tissue were all significantly increased compared to those in adjacent normal mucosa tissue samples (all P<0.001, Fig. 4), suggesting that high NF-κB mRNA levels might be positively correlated with IL-6 mRNA levels.

### Protein levels are markedly upregulated in gastric cancer tissue

**Figure 5:**
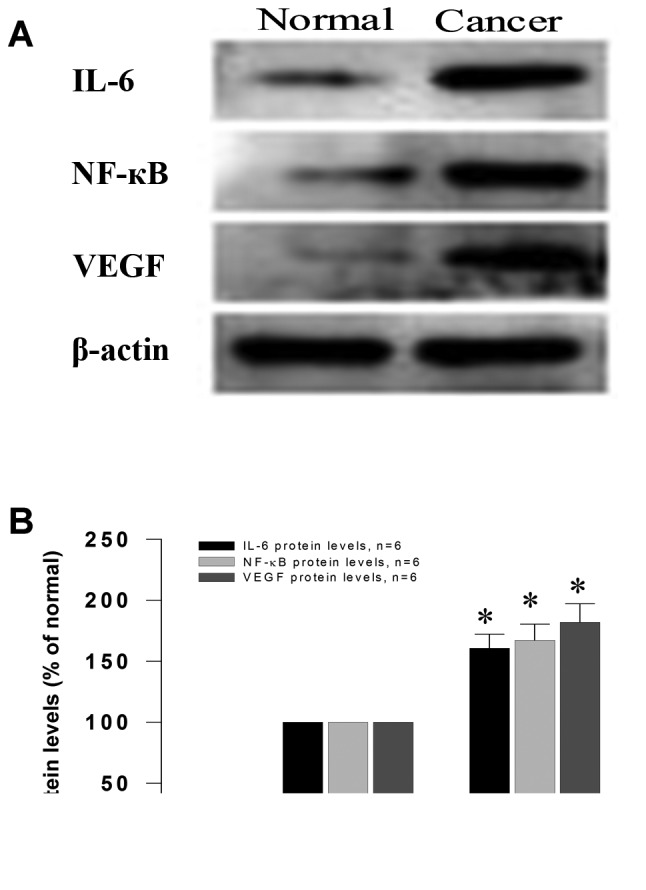
Protein expression of IL-6, NF-κB, and VEGF was determined by western blotting (A). The protein levels of IL-6, NF-κB, and VEGF increased significantly in gastric cancer tissue compared with those in the adjacent normal tissue (^*^P<0.05). β-actin was used as a loading control. Quantitative analysis showed the protein expression levels of IL-6, NF-κB, and VEGF in the two groups (B). ^*^P<0.05, compared with normal tissues, n=6.

We further explored the protein expression of IL-6, NF-κB and VEGF in gastric cancer tissue according to western blotting. As we expected, a single band was performed using each antibody (Fig. 5A) and the protein production levels of IL-6, NF-κB and VEGF in human gastric cancer tissue were all clearly upregulated compared to those in the adjacent normal mucosa tissue (all P<0.001, Fig. 5B). IL-6, NF-κB and VEGF increased significantly in gastric cancer tissue, suggesting that high protein levels of NF-κB might be positively correlated with IL-6 protein levels.

## Discussion

NF-κB, discovered in 1986, binds to the enhancer region of the κB chain of immunoglobulin as a nuclear factor in B cells. Constitutive activation of NF-κB has been found in the majority of tumor cell lines, including solid and hematologic tumors (14). Proliferation of most tumor cells depends on constitutive activation of NF-κB, as inhibition of NF-κB leads to abrogation of proliferation (15). Pro-inflammatory cytokines such as TNF, IL-1 and IL-6, all regulated by the NF-κB pathway, have been shown to be overexpressed in colitis, gastritis, or hepatitis. IL-6, whose expression is regulated by NF-κB, has been implicated in the oncogenesis process by inducing proliferation of multiple myeloma cells (16). In gastric carcinoma, IL-6 induces VEGF expression by increasing angiogenesis (17), and may be a marker of tumor angiogenesis and disease status (18,19).

In the present study, we showed that activation of NF-κB correlates with IL-6 in human gastric cancer tissues. These data indicating a role for NF-κB and IL-6 are supported by studies in patients with gastric cancer. In particular, expression of IL-6 mRNA in gastric mucosa related to the level of gastric mucosal inflammation cytokine (20,21). Serum levels of IL-6 and TNF-α were significantly higher in patients with gastric cancer than gastritis (22). IL-6 plays a key role as a prognostic factor in gastric cancer invasion and lymph and/or hepatic node metastasis (13), and consistent with our results, in a series of gastric cancer patients, high IL-6 serum levels predict a shorter survival.

NF-κB mediates the expression of most gene products that play key roles in cell survival, angiogenesis and immune responses. One of the gene targets of NF-κB is IL-6. The IL-6 promoter involves at least four transcription factor binding sites, the IL-6-NF-κB regulatory site is one of them. Although the transcriptional regulation of IL-6 expression appears to be very complex, including multiple transcription factors and signaling pathways, NF-κB may play a crucial role in the expression of IL-6 in gastric cancer. In numerous cells, activation of NF-κB is responsible for the inducible production of the proinflammatory cytokines, involving IL-1b, IL-6, IL-8, and TNF-α (23,24). These studies are consistent with our results.

In the present study, we found that IL-6 expression was significantly associated with NF-κB, and both were found overexpressed in human gastric cancer; a weak expression was found in adjacent normal mucosa. Since our results indicate a differential expression of IL-6 and NF-κB in gastric cancer tissue and adjacent normal mucosa, this may suggest that the expression pattern of the IL-6-NF-κB signal pathway may be linked to the development of gastric cancer.

NF-κB inhibition does not completely prevent cancer pathogenesis, as cytokines could also promote tumorigenesis via alternative pathways (25). Therefore, investigations of other molecular pathways may provide further insights into chronic inflammation-induced tumorigenesis and targeted cancer therapy.

## Figures and Tables

**Table I:** Comparison of plasma cytokine levels of IL-6 and TNF-α in gastric cancer patients.

Variable	No.	IL-6 (ng/l)	TNF-α (ng/l)
Pre-operative	30	279.2±56.7^a^	315.4±60.7^a^
Post-operative	33	183.2±39.5^a^,^b^	236.5±31.8^a^,^b^
Normal	35	38.9±11.2	53.5±17.6

Data are means ± SD. Compared with the pre-operative group, P<0.01, ^a^P<0.05. Compared with normal group, P<0.01, ^b^P<0.05.
